# α-Synuclein Fibrils as Penrose Machines: A Chameleon in the Gear

**DOI:** 10.3390/biom12040494

**Published:** 2022-03-24

**Authors:** Francesca De Giorgi, Vladimir N. Uversky, François Ichas

**Affiliations:** 1 CNRS, Institut des Maladies Neurodégénératives, UMR 5293, 33076 Bordeaux, France; 2 Institut des Maladies Neurodégénératives, UMR 5293, Université de Bordeaux, 33076 Bordeaux, France; 3 Department of Molecular Medicine, Morsani College of Medicine, University of South Florida, Bruce B. Downs Blvd., MDC07, Tampa, FL 33612, USA

**Keywords:** α-Synuclein, amyloid, fibril, strain, intrinsically disordered protein, Parkinson’s disease, prion, PrP, sup35, seeding, templating, self-replication

## Abstract

In 1957, Lionel Penrose built the first man-made self-replicating mechanical device and illustrated its function in a series of machine prototypes, prefiguring our current view of the genesis and the proliferation of amyloid fibrils. He invented and demonstrated, with the help of his son Roger, the concepts that decades later, would become the fundamentals of prion and prion-like neurobiology: nucleation, seeding and conformational templating of monomers, linear polymer elongation, fragmentation, and spread. He published his premonitory discovery in a movie he publicly presented at only two conferences in 1958, a movie we thus reproduce here. By making a 30-year-jump in the early 90’s, we evoke the studies performed by Peter Lansbury and his group in which α-Synuclein (α-Syn) was for the first time (i) compared to a prion; (ii) shown to contain a fibrillization-prone domain capable of seeding its own assembly into fibrils; (iii) identified as an intrinsically disordered protein (IDP), and which, in the early 2000s, (iv) was described by one of us as a protein chameleon. We use these temporally distant breakthroughs to propose that the combination of the chameleon nature of α-Syn with the rigid gear of the Penrose machine is sufficient to account for a phenomenon that is of current interest: the emergence and the spread of a variety of α-Syn fibril strains in α-Synucleinopathies.

With a sequence of four seminal papers published in 1993 [1], 1994 [2] and 1995 [3,4], Peter T. Lansbury was, with his group, the first to reveal the striking homologies that exist between the properties of α-Synuclein (α-Syn) [5] and the prion protein [6,7,8], and to propose a unifying conformational templating model in which seeded polymerization would fuel the spread of their respective pathological assemblies [3] (Figure 1). The idea of this model probably also stemmed from another concomitant finding from his group: α-Syn is an intrinsically disordered protein (IDP) [9]–a status they called “natively unfolded”– and thus particularly capable of accomodating conformational templating [10]. This was later formulated as a “protein-chameleon” concept proposed by one of us in 2003 [11]. As shown for prion, an intriguing consequence of the model was that the product of the seeded polymerization process, i.e., a serially-amplified copycat of the initial seed [12], could diffuse and spark an autocatalytic spread proceeding as long as monomers would remain available to feed it [13]. As to the nature of those spreading seeds, the debatable presence of bona fide amyloid fibrils in prion-infected brains [14] probably contributed to then convince Lansbury and his colleagues that the long α-Syn amyloid filaments found in neuronal inclusions in Parkinson’s disease [15] could not be the culprits [16]. He gave up the idea that they could represent the self-replicating assemblies fitting his unifying seeding–templating model: the α-Syn fibrils would rather represent occasional dead-end pacified structures, while as a versatile IDP, α-Syn should undergo nucleation and proliferate under the form of much smaller neurotoxic amyloid-like assemblies [16], more like prions [4]. However, what if the long α-Syn fibrils spontaneously break into pieces after some time of elongation instead? Could this reconcile fibrils with the spread?

Most interestingly, the notion that amyloid fibrils could act as amyloidogenic seeds was proposed very early regarding insulin [17]. This was experimentally confirmed in 1991 when it was shown that fibril fragments artificially generated by sonication were indeed behaving as seeds [18]: the fragments put in the presence of their corresponding soluble protein monomers (apolipoprotein A-II in this study) served as templates to the *de novo* formation of fibrils by progressive elongation taking place at both ends of the growing seeds. These observations are supportive of the idea that, in the model of Lansbury, a possible delayed fragmentation of the long α-Syn fibrils could indeed constitute the key to self-amplification and auto-catalytic spread, granting the whole process with an ability to propagate like an auto-wave in a distributed chemical [19] or biological [20,21,22] system.

Though representing quite rigid structures, the general idea that amyloids could indeed get fragmented in living cells and that this could represent a key step for their replication and spread was first proposed in 1998 regarding the sup35 prion in yeast [23]. In this model, also reviewed in [24], a chaperone attacks the amyloid assembly in an attempt to refold the monomers, but by doing so also produces breaks that generate fibrils fragments acting as new seeds, such as in the artificial fibril sonication experiment discussed before [18]. This “extrinsic” model of fragmentation, as well as its role in the propagation of α-Syn assemblies, has successfully been recapitulated in the nematode very recently [25,26].

Another mechanism of fibril fragmentation is “intrinsic” and structurally encoded, i.e., it depends on the details of the elemental amyloid fold imposed to the monomers during templating [27]. Obeying this “stability encoding”, elongating fibrils can undergo self-splitting events accelerated by stirring [28]. The fact that the replication characteristics of different prion strains could reflect different stability encodings was first documented in yeast for the sup35 prion in 2004 [29]. Regarding α-Syn, strain-dependent frangibility was demonstrated in vitro in 2010 [30]: it was found that depending on the strain, the persistence length of the fibrils could differ by as much as three orders of magnitude. The strain-dependence of the α-Syn fibril frangibility and its inheritance through seeding/templating was later observed in living neurons [31].

The main and key consequence of this intrinsic mechanism of fibril fragmentation is that its integration into the model of Lansbury [1] completes the picture of a full self-replicating cycle, in which the self-replicating unit is properly the fibril fragment seed.

This picture together with its entire sequence of events had been theorized and mechanically modelized by Lionel Penrose almost 40 years before, in 1957 [32]. However, Lionel Penrose’s intuition was left unnoticed in the amyloid field. A possible reason is that the short note he wrote in *Nature* with his son Roger was not very self-explanatory and appealing. In addition, John Griffith, who is considered as the precursor of the prion concept, only marginally mentioned Penrose’s work in his own *Nature* paper published 10 years later and where he proposed his self-replication models of scrapies [7]. However, Lionel Penrose was conscious that his discovery was not properly served by a printed note and decided to also demonstrate his machines at work in a striking movie (https://doi.org/10.5281/zenodo.6208683) (Supplementary Video S1). This movie was publicly shown in only two instances in 1958: at a Research Film show in Scotland [33] and then in Canada at a genetics conference held at the MacGill University [34] (Supplementary Figure S1). Unfortunately, the concepts it was illustrating were so at the forefront and its broadcasting so difficult that it did not meet the relevant public of biologists or of supramolecular chemists who could have understood its significance, even if some could probably have already been prepared for this in 1957 [17]. Figure 2 shows the reproduction of a local newspaper’s columns covering the presentation at the MacGill University conference and summarizing the discussion the journalist had with Lionel Penrose (anecdotally, note the reference to his son Roger presented as “a mathematician and an internationally famed chess-player”: he is the theoretical astrophysicist who explained the origins of Black Holes and won the Nobel prize in 2020).

It is very difficult not to interpret the coverage reproduced in Figure 2 as a visionary discussion about prions or other self-replicating amyloids. The same remark also applies of course to the original movie (https://doi.org/10.5281/zenodo.6208683) (Supplementary Video S1), which will certainly leave speechless all the colleagues who personally fibrillized amyloids with their own hands starting from recombinant monomers, and who seeded their preparations to study fibrillization kinetics, amyloid strains, etc. not to mention that the process of self-replication is also obtained here by stirring and shaking the monomers. In the movie, Lionel Penrose progressively illustrates how the combination of different simple laws can bring inert monomers to assembly and “life” (self-replication): (i) the process of seeding; (ii) the linear templated polymerization, and (iii) the division of the polymer releasing new seeds. It is almost unbelievable that the notion of seed strains resulting in specific polymer assembly architectures, as well as the self-replication of homodimer seeds through their templating into intertwined linear polymer assemblies, are also shown at work.

One important point highlighted by Lionel Penrose is that in an unshaken distributed system the process would have taken place similarly, on a time scale only defined by diffusion and Brownian motion. In other words, in the case of a localized introduction of a seed in a resting distributed system of monomers, this would have caused the spatial spread of seeded polymerization and replication throughout the system, just like the propagation of a traveling wave in an excitable medium [19,20,21,22,35,36,37]. This is due to the fact that once the process is initiated in a localized spot by a seed, the local diffusion of the neoformed seeds causes them to encounter nearby monomers and recruit the latter in an “offset” templated polymerization process, creating new seeds, that diffuse in turn, and so on until the polymerization wavefront has swept all the volume occupied by the distributed monomer system. If one now equates the brain to a simple diffusion volume (i) homogeneously filled with α-Syn or physiological prion proteins, and (ii) fully partitioned into adjacent neurons with their plasma membrane offering limited resistance to the diffusion of seeds, one would then expect that a localized intracerebral seeding event would spark the spread of an aggregation wavefront traveling from one neuron to another and reaching brain regions distant from the original seeding site. After decades, the biological relevance of this striking prediction was demonstrated by brain graft experiments conducted in living mice regarding prions [13], and for α-Syn, in retrospective and therapeutic graft follow-up neuropathological studies conducted on the brains of deceased Parkinson’s disease patients [38,39,40] and eventually in intracerebral seeding experiments in mice [41,42].

It was pointed out that the intrinsically disordered nature of α-Syn (and, as a matter of fact, many other amyloidogenic proteins) provides important means for a generation of the multiple strains of amyloidogenic seeds and resulting fibrils. This mechanism is based on the ability of the monomeric forms of this protein to accommodate different structural ensembles depending on their environment. In fact, based on a series of studies conducted in the group of Anthony L. Fink and Vladimir N. Uversky in the early 2000s, where the effects of various environmental conditions on structural properties and aggregation propensity of α-Syn were analyzed, the concept of “protein-chameleon” was proposed in 2003 [11]. Here, the comprehensive experimental analysis suggested that the conformational equilibrium of the dynamic structural ensemble of this protein can be shifted to favor the formation of various partially folded forms. For example, a decrease in pH or an increase in temperature transformed α-Syn into a partially-folded amyloidogenic conformation [43]. Similarly, species with the increased aggregation potential were induced in this protein by pesticides and herbicides [44,45], certain metal ions [46,47], point mutations associated with familial forms of PD [48,49], natural osmolyte trimethylamine-N-oxide (TMAO, where low to moderate TMAO concentrations promoted the formation of the amyloidogenic partially-folded monomer, whereas high concentrations of this osmolyte induced tightly-folded α-Syn species forming specific off-pathway oligomers with high helical content) [50], and various organic solvents (where simple and fluorinated alcohols induced complex, multistage folding of this protein, with the common first stage being the formation of a partially folded intermediate with an enhanced propensity to fibrillate) [51]. This environment-driven structural polymorphism of the monomeric states of α-Syn and other amyloidogenic IDPs defines their ability to form a multitude of different oligomeric and aggregated forms [52].

Oligomerization was shown to stabilize partially folded conformations of α-Syn both in vitro and in cytosolic preparations [53]. Fibrillation of this protein was accelerated by its interaction with heparin and other glycosaminoglycans [54], binding to different unstructured polycations (spermine, polylysine, polyarginine, and polyethyleneimine) [55], interaction with histones [56], in the presence of moderate TMAO concentrations [50] or low concentrations of organic solvents [51], and in the crowded milieu [57]. On the other hand, fibrillation was inhibited by interaction with β- and γ-synucleins [58], methionine oxidation [59], nitration [60], high concentrations of TMAO [50], or organic solvents [51]. Depending on the environmental conditions, these stable soluble oligomers were shown to have either a mostly α-helical conformation or instead β-structure-enriched domains [50,51]. Furthermore, combinations of different environmental factors showed different effects on α-Syn fibrillation. For example, metal ions and pesticides acted synergistically on the fibrillation rates of this protein [47], whereas some metal ions triggered fibrillation of methionine-oxidized form of α-Syn, which cannot fibrillate alone [61].

Obviously, all these observations provided rich ground, from which the protein chameleon concept originated. Subsequent research of many groups further supported the remarkable complexity of this protein, its structural polymorphism, binding promiscuity, multifunctionality, and multipathogenicity, where one protein can be associated with the pathogenesis of various diseases. Recently, it was pointed out that the reconciliation of numerous and often contradictory results of multiple studies dedicated to this protein (as of 16 February 2022, PubMed contained more than 42,000 α-Syn-centric papers) can be reached using the proteoform concept [62]. It was also pointed out that “the intrinsically disordered nature of the protein, which defines the structural polymorphism of the conformational ensemble of its monomeric form, bestows multifunctionality upon α-Syn. Logical consequences of this structural heterogeneity and multifunctionality of a monomeric protein are the structural polymorphism of aggregated states and a “spectrum of dysfunctions” that define a range of diseases” [62].

Combining these considerations with the afore-mentioned Penrose concepts adds further levels of complexity to the molecular mechanism of fibril propagation. In fact, considering α-Syn in the framework of the mechanisms of the Penrose machines, it is likely that its “extreme” IDP status [9] underlies not only (i) the possibility to achieve a variety of possible seed conformations giving birth to as many fibril strains and self-replicating assemblies, but also (ii) to tweak the conformational templating gear by occasionally getting incorporated into the growing polymer as incompletely or differently folded monomers, as recently proposed for prions [63]. The combination of the occurrence of such templating errors (conformational mismatches [63]) with a high replication rate by fibril division could be at the origin of the emergence of new strains while an amyloid proliferation is already initiated. Indeed, as detailed before, depending on the physico–chemical context, α-Syn can transitorily acquire “prefolded” conformations, and in particular, conformations with partial beta-strand structurations [43]. These monomeric forms have previously been considered as intermediates in the slow process of seed nucleation, but alternatively, they might be quickly recruited during the fibril elongation process and deviate the supramolecular architecture “from the initial plans”. This would affect the self-replicating characteristics of the fibrils, and result in the release of a new strain of seeds during fibril division. In addition, it is worth noting that actively replicating fibrils could also catalyze/promote secondary nucleations taking place at their outer surface without templating [64], i.e., easily leading to novel fibril strains.

These possibilities are particularly intriguing because they would equate α-Syn fibrillar amyloids to self-replicating structures capable of adaptative molecular evolution: indeed, because of their chameleon nature, it would be sufficient that only a very few α-Syn monomers transitorily adapt to the milieu to have long-lasting consequences: their direct incorporation into growing fibrils would by-pass the intractable kinetic limitation of nucleation and immediately amplify the adaptative event by templated elongation on this “new model” [63], causing the emergence of a novel “adapted” seed strain after fibril division. The same would hold true for fibril-catalyzed secondary nucleations taking place without any templating pressure [64].

While the adaptative molecular evolution of α-Syn fibrils could sound like a blatant overstatement, previous experimental work performed both in vitro and in vivo strongly supports this view instead [65,66]. Indeed, in both studies, it was found that environmental information gets imprinted in the supramolecular architecture of the replicating α-Syn fibrils, and was eventually at the origin of the formation of novel strains capable of perpetuating their new “informed” fold. This is of key importance because the novel “adapted” fibril strains were endowed with novel morphological and functional or pathophysiological characteristics.

The chameleon in the gear of the Penrose machine thus offers a framework to understand the diversification of pathological α-Syn amyloid strains during disease [66,67]. It also helps to contemplate self-replicating amyloids in general, and the role they might have played in the primordial soup (Figure 2) [68,69].

## Figures and Tables

**Figure 1:**
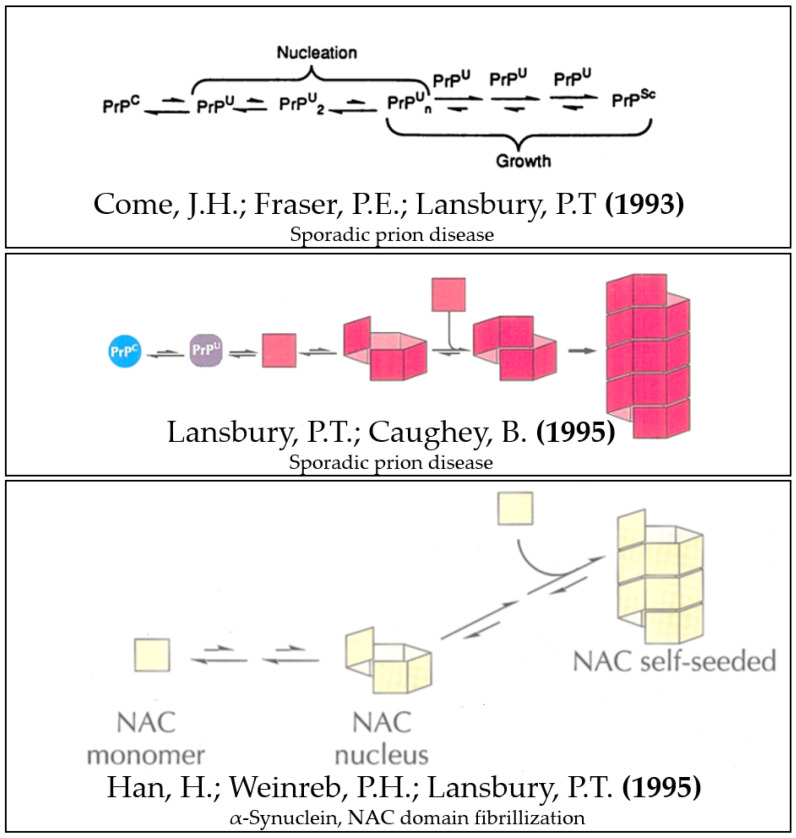
Lansbury’s unified seeding/templating model for sporadic prion disease and α-Synuclein fibrillization. Modified and composed from figures of references [1,3,4].

**Figure 2:**
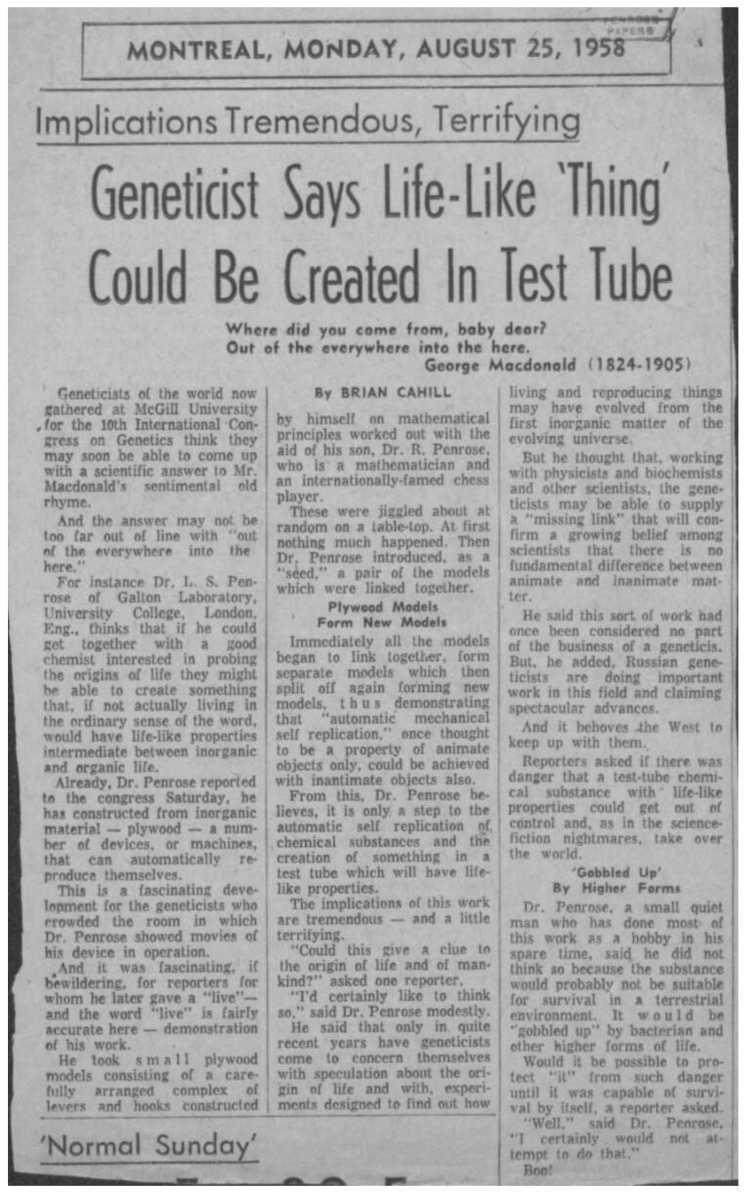
Coverage of the Lionel Penrose presentation at the MacGill University conference by the journalist Brian Cahill for the local Newspaper “Montreal Star”.

## Supplementary Materials

The following supporting information can be downloaded at: https://www.mdpi.com/article/10.3390/biom12040494/s1, Figure S1: ”Automatic Mechanical Self-Replication” by L.S. Penrose, Original Manuscript of the Movie Abstract; Video S1: Automatic Mechanical Self-Replication” by L.S. Penrose.
