# Supplementary material for: α-Synuclein Fibrils as Penrose Machines: A Chameleon in the Gear
Source: Biomolecules. 2022 Mar 24;12(4):494. doi: 10.3390/biom12040494 (PMC9029340; doi:10.3390/biom12040494)
Supplement: Supplementary file 1 [file biomolecules-12-00494-s001.zip › biomolecules-1628145-supplementary S1.pdf]

Supplementary Data:

PENROSE PAPERS

X INTERNATIONAL CONGRESS OF GENETICS

AUTOMATIC MECHANICAL SELF REPLICATION

Motion Picture; session C - 8, 10 a.m. Saturday, 23rd August. 1958.

L. S. Penrose, Galton Laboratory, University College, London, England.

Theoretical discussion of the principles of automatic self replication was initiated by von Neuman in 1951. The conclusion was drawn then that construction of an automation, which could build replicas of itself, was possible. Many different parts of unspecified complexity would be needed, and energy would have to be supplied from outside.

An elementary practical solution of the problem was described by L.S. & R. Penrose in 1957. In this model, the material consisted of solid units of only two kinds which were subjected to agitation in a horizontal track. When a linked pair was introduced as a seed, it caused the formation of other linked pairs of the same type.

Soon afterwards, systems with more life-like properties were constructed on similar principles. For example, automatic replication, which involved the growth and eventual partition of the original seed, was demonstrated.

The progress of these ideas, from very simple designs capable only of mimicking crystal formation, to analogues of mitosis is sketched in the motion picture. Subsequently, models exhibiting recombination have been built. The accurate automatic replication of chains, unlimited in complexity, has been described in papers now in the press.

References:--

1. Von Neumann, J. (1951) Cerebral Mechanisms in Behaviour. New York & London: McGraw Hill.
2. Penrose, L.S. & Penrose, R. (1957) A Self-reproducing analogue. Nature, 179, 1183.
3. Penrose, L.S. Automatic Mechanical Self-Reproduction. New Biology (in the Press).
4. Penrose, S. Mechanics of Self-Reproduction. Annals of Human Genetics (in the Press).

Supplementary Figure S1: Abstract sent by L.S. Penrose to participate to the 10th International Congress of Genetics, August 20-27 1958, McGill University, Montreal
